# Determinants of Catastrophic Dental Health Expenditure in China

**DOI:** 10.1371/journal.pone.0168341

**Published:** 2016-12-15

**Authors:** Xiangyu Sun, Eduardo Bernabé, Xuenan Liu, Jennifer Elizabeth Gallagher, Shuguo Zheng

**Affiliations:** 1 Department of Preventive Dentistry, Peking University School and Hospital of Stomatology, National Engineering Laboratory for Digital and Material Technology of Stomatology, Beijing Key Laboratory of Digital Stomatology, Haidian District, Beijing, People’s Republic of China; 2 King’s College London Dental Institute at Guy’s, King’s College and St. Thomas’ Hospitals, Population and Patient Health Division, London, United Kingdom; Institute of Postgraduate Medical Education and Research, INDIA

## Abstract

This study explored catastrophic health expenditure in China, due to out-of-pocket payments for dental care, and its associated individual- and contextual-level factors. We pooled data from 31,566 adults who participated in the third National Oral Health Survey with province-level data from different sources. We defined catastrophic dental health expenditure (CDHE) as payments for dental services and/or medication for dental problems during the last year that exceeded the 10% and 20% of the household income. The association of individual and contextual factors with catastrophic dental health expenditure was evaluated using two-level logistic regression models with individuals nested within provinces. Socioeconomic position (education and household income), household size and dental status (pain in teeth or mouth and number of teeth) were the individual-level factors associated with CDHE among the full sample of participants; and, also, among those who used dental services in the past year. Greater gross domestic product per capita was the only contextual factor associated with CDHE, and only at the lower income threshold. This study shows that out-of-pocket expenses for dental services may put a considerable, and unnecessary, burden on households’ finances. Our findings also help characterise those households more likely to face catastrophic expenditure on health if they have to pay for dental services.

## Introduction

The World Health Organization (WHO) has called upon governments to move towards universal health coverage [1]. Such commitment requires that everyone receives the health services they need, without exposure to financial hardship [1,2]. Financial protection is an underlying principle of universal health coverage and, as such, it receives considerable attention in health financing policy [3]. The WHO and World Bank Global Monitoring Framework [4,5], have proposed two population metrics to track levels of financial protection, namely the incidence of catastrophic health expenditure (CHE) and impoverishment due to out-of-pocket expenses for health services. CHE reflects health spending that exceeds a certain threshold of the household’s disposable income whilst impoverishment reflects health spending that pushes households below the poverty line [6,7].

Recent studies have shown that poorer and more unequal countries are more likely to have more households facing CHE [8,9]; and that households in rural areas, living in low income, having young children and/or older adults and lacking health insurance were more likely to incur in CHE [8,10,11]. In addition, the use of specific health services, such as inpatient care, prescription drugs and visits to traditional healers, may lead to CHE [12–15]. There is some evidence that out-of-pocket payment for dental services was associated with CHE. In Korea, CHE was more common among households that used dental services (24.6%) than among those that did not use those services (7.8%), although no adjusted results were reported [13]. In Iran, households that used dental services in the last four weeks were four times more likely to incur CHE than those not using those services, after controlling for household’s socioeconomic status and composition, health insurance and use of inpatient and outpatient services [16]. A recent multilevel study across 41 developing countries showed that catastrophic dental health expenditure (CDHE) was more prevalent in more economically developed countries and better-off, urban and larger households [11]. Evidence from countries at different stage of economic development is still needed.

The People’s Republic of China is going through a large socioeconomic transformation that has shifted the disease burden towards chronic conditions [17,18]. The 5-year Chinese health reform, that started in 2003, focused on equitable access (especially for people in rural areas) through the expansion of social health insurance [19,20]. China’s achievement of moving towards universal health insurance has been remarkable in terms of the scale of coverage expansion and the speed of expansion over the past two decades [19,21]. However, only a small portion of dental care is covered by insurance schemes [22]. This has led to the proliferation of private dental clinics in order to satisfy the ever-increasing need and demand for dental care [23], and thus out-of-pocket spending on dental care. This study relates to the early period of health reforms in China, with a view to setting baseline evidence for monitoring purposes as new national data become available.

The aim of this study was to determine the level of CDHE in China; and the individual- and contextual-level factors associated with CDHE.

## Methods

### Data source

This cross-sectional study pooled individual- and province-level data from various sources. Individual-level data were from the 3^rd^ National Oral Health Survey of China (2005), which covered the four World Health Organization (WHO) index ages (5-, 12-, 35- to 44-, and 65- to 74-year-olds). All 31 provinces of Mainland China participated in the survey, except for Tibet where administrative authorisation was not obtained. Participants were selected using multistage stratified cluster sampling. Each province was divided into urban and rural areas; urban areas were classified into three strata by population size, whereas rural areas were classified into three strata by Gross Domestic Product (GDP). One city or county was randomly selected from each stratum. Hence, three cities from urban areas and three counties from rural areas were selected from each province. For the next level of sampling, three streets or townships were randomly chosen from every city or county, respectively. Two residents’ committees in these streets (or two villages in townships) were recruited randomly from the list of residents provided by each residents’ committee. At each survey station, 20 working-age and 20 senior adults were recruited randomly. A target sample of 720 participants in each age group was initially set per province, for a total of 21,600 people nationally. Among 35-44-year-olds, 23,538 participated in clinical examinations and 23,522 completed the questionnaire. Among 65-74-year-olds, 23,415 were clinically examined and 12,893 completed the questionnaire (only half of the senior sample was invited to complete the questionnaire). A total of 31,566 participants (21,015 adults and 10,551 older adults) had complete data in all relevant variables and were included in the present analysis (representing 87% of the total number of participants in the national survey).

### Variables selection

Out-of-pocket expenditure for healthcare is defined as ‘catastrophic’ if it exceeds a certain threshold in a given period [6]. The threshold represents a predefined proportion of household income/expenditure, which can vary from 5% to 40% [24,25]. Lower thresholds are typically used when total income/expenditure is in the denominator while higher thresholds are used when food expenditure is subtracted from the denominator [7]. The latter approach assumes that food and health care expenditure are not substitutes [6]. Since both food and health expenditures are necessities, we did not subtract food expenditure from total income [24]. Participants were asked to report how much money they had spent on the following: (i) dental treatments (any dental procedure for disease treatment or aesthetic reasons); and (ii) medication for dental problems (drugs or traditional medicine regardless of whether it was prescribed or self-medicated) in the last 12 months. Indirect costs (such as transportation or loss productivity) were not included in the responses. Dental health expenditure was calculated as the sum of responses to the two items and defined as catastrophic using two progressive cut-off points, namely 10% and 20% of total household income. Using a higher threshold (40%) yielded a very low prevalence of CDHE (0.2%) and yielded unreliable estimates from regression models.

A number of individual- and province-level factors were included in the analysis as potential determinants of CDHE. Demographic factors were sex, age, ethnicity and place of residence (urban or rural). Participants’ ethnicity was self-assigned using a list of officially recognised ethnic groups in China, and responses classified as ‘Han’ or ‘other’ ethnic minority group. Socioeconomic position (SEP) was measured using participants’ education and household income. Participants reported their total number of years of full-time education, and responses were re-grouped in line with national cut-off points: primary school (0–6 years), junior middle school (7–9 years), senior middle school (10–12 years) and higher education (13+ years). Participants were also asked to provide an estimate of their annual household income with no pre-set categories. Income data were equivalised using the Luxembourg Income Study scale to account for family size [26,27]. This involved dividing the total household income by the square root of the number of individuals in the family [26]. After equivalisation, household income in Chinese Yuan was categorised into tertiles: 1^st^ tertile/low (<3,530), 2nd tertile/medium (3,530–8,660) and 3rd tertile/high (>8,660). Household size was measured as the number of adults and children in the family and recoded into three groups by size: 1–2, 3–4 and 5+ members. Participants also reported whether they had any kind of dental health insurance plan and whether they have experienced pain in their teeth or mouth during the last 12 months (never, rarely, sometimes or often).

Dental clinical examinations were carried out with participants seated on a chair, and using artificial light, plane mouth mirrors and standard WHO CPI probes. Unified training sessions were provided to over 200 survey examiners in Kunming city, Yunnan, before the national survey began. All teeth, excluding third molars, were examined. Dental caries was diagnosed according to the WHO criteria [28]. For reliability assessment, duplicate examinations were conducted during the main survey. Five percent of the participants were re-examined to calculate inter-examiner reliability. The Kappa score was 0.89 among 35-44-year-olds and 0.93 among 65-74-year-olds. Only the number of teeth was used to indicate participants’ dental status as using other clinical measures (such as dental caries experience or periodontal disease) would have restricted the analysis to dentate participants.

Province-level data were gathered from different national and international sources [29–31] matching the survey year as closely as possible. Macroeconomic factors were income inequality and average income at province level. Income inequality was measured using the Gini coefficient, expressed as a percentage where higher values indicate greater inequality, for the period 1985–1995 [29]. More recent income inequality data are not available due to the lack of comprehensive income surveys in China [32]. Average income was measured in terms of GDP per capita in 2005, expressed in thousand Yuan [30]. Health financing was measured in terms of public health expenditure, which refers to expenditure on health care incurred by government finance and is expressed as a proportion of total government public expenditure in 2005 [31].

### Statistical analysis

Post-stratification weights were used to adjust for differences in the age-by-sex-by-ethnicity-by-province distribution between the sample and the general population in the 30 provinces involved in the study, in line with the 5^th^ National Demographic Census in 2000. Analyses also took into account the complex survey design (stratification and clustering) to produce corrected standard errors.

Two analytical samples were used in this study, namely the full sample of adults and the subgroup who visited the dentist the year prior to the survey (henceforth referred to as service users). Restricting our analysis to services users would have not captured the full of extent of CDHE since we would have excluded those who might have incurred some expenditure on medicines for dental relief without visiting a dentist. We first present the composition of each sample according to individual- (sex, age, ethnicity, place of residence, education, income, household size, dental insurance, number of teeth and pain in teeth or mouth) and province-level factors (GDP per capita, Gini coefficient and public health expenditure). The impact of missing data was evaluated comparing the profiles of participants with complete data and those excluded due to missing values using the Chi-square test. The prevalence of CDHE (at 10%- and 20%-thresholds) was then compared by individual-level factors using the Chi-square test.

A two-level random-intercepts and fixed-slopes model structure, with individuals nested within provinces was fitted, using binary logistic regression as CDHE was an uncommon (i.e. less than 10%) dichotomous outcome. Odds ratios (OR) were therefore reported. The fixed- and random-parameter estimates for the multilevel models were calculated using marginal quasi-likelihood (MQL) procedures as implemented in MLwiN 2.29. These analyses were conducted using the unweighted samples as the level-2 weights, needed to compensate for the unequal probability of selection of level-2 units [33,34], were not available. Non-weighted analyses were appropriate as our focus was on tests of association rather than deriving nationally representative estimates. More importantly, minimal differences have been observed in estimates and standard errors from weighted and unweighted multilevel regression [35]. The association of each individual- and province-level factor with CDHE (at 10%- and 20%-thresholds) was assessed in crude and adjusted models. The adjusted model included all individual- and province-level factors as explanatory variables.

## Results

The characteristics of the two study samples are shown in Table 1. The mean number of participants per province was 1,052 (range: 789–1,542). Male, younger and more educated adults, together with those living in urban areas and larger households, and those with more teeth, were more likely to be included in the sample. The mean annual household income was 14,543 Chinese Yuan (Standard Deviation [SD]: 24,684; range: 9 to 900,000) in the full study sample and the mean expenditure for dental care among those who paid for those services was 283 Chinese Yuan (SD: 2,600; range: 1 to 200,000).

**Table 1:** Characteristics of the sample of Chinese adults (n = 31,566) and of the subsample of participants who used dental services in the last year (n = 5,511)

Factors		All adults^a^		Service users^a^	
**Level 1: Individual**					
*Sex*	Men	16059	52.0%	2607	48.8%
	Women	15507	48.0%	2904	51.2%
*Age*	35~44 years	21015	67.2%	3416	62.3%
	65~74 years	10551	32.8%	2095	37.7%
*Ethinicity*	Han	28333	93.6%	5019	94.4%
	Ethnic minority group	3233	6.4%	492	5.6%
*Place of residence*	Urban	16222	40.1%	3262	47.0%
	Rural	15344	59.9%	2249	53.0%
*Education*	Up to primary school	12630	42.7%	1997	39.8%
	Junior middle school	9506	31.0%	1549	29.2%
	Senior middle school	6044	17.4%	1148	18.7%
	Higher education	3386	8.9%	817	12.3%
*Income*	1st tertile (Low)	10509	37.1%	1497	31.8%
	2nd tertile (Medium)	10375	33.1%	1739	32.1%
	3rd tertile (High)	10682	29.9%	2275	36.0%
*Household size*	1~2 members	6801	21.7%	1288	23.2%
	3~4 members	18011	56.8%	3126	56.3%
	5+ members	6754	21.4%	1097	20.5%
*Dental insurance*	Not insured	25171	83.1%	4177	79.5%
	Insured	6395	16.9%	1334	20.5%
*Pain in teeth or*	Never	15483	49.7%	926	16.7%
*mouth*	Rarely	5949	18.4%	1428	25.2%
	Sometimes	7199	23.0%	2162	39.9%
	Often	2935	8.9%	995	18.1%
*Number of teeth*	No teeth	666	2.1%	76	1.5%
	1–19 teeth	3356	10.9%	757	14.1%
	20+ teeth	27544	87.0%	4678	84.4%
**Level 2: Province (n = 30)**					
*GDP per capita*, *thousand yuan*		16.2 ± 8.9		16.6 ± 9.5	
*Gini co-efficient*, *%*		20.5 ± 2.0		20.4 ± 2.0	
*Public health expenditure*, *%*		4.0 ± 0.8		4.1 ± 0.8	

^a^ Counts are unweighted.

The overall prevalence of CDHE was 1.4% (95%CI: 1.2–1.6%) and 0.5% (95%CI: 0.4–0.7%) at the 10% and 20% income thresholds respectively. As expected, the prevalence of CDHE was higher among service users: namely 8.1% (95% CI: 6.9–9.3%) at 10%; and 3.2% (95% CI: 2.5–4.2%) at 20% threshold. Significant differences in the proportion of CDHE were found by all individual-level factors except for sex and ethnicity both in the full sample and among service users (Table 2).

**Table 2:** Catastrophic dental health expenditure at different income thresholds among all adults and those who used dental services in the last year, by individual-level factors

Factors		All adults (n = 31,566)		Service users (n = 5,511)	
		At 10%	At 20%	At 10%	At 20%
*Sex*	Men	1.3%	0.5%	7.9%	3.2%
	Women	1.5%	0.6%	8.2%	3.2%
	*P value*^*a*^	*0*.*112*	*0*.*331*	*0*.*417*	*0*.*505*
*Age*	35~44 years	0.9%	0.4%	5.9%	2.3%
	65~74 years	2.2%	0.9%	11.5%	4.8%
	*P value*	*<0*.*001*	*<0*.*001*	*<0*.*001*	*<0*.*001*
*Ethnicity*	Han	1.4%	0.5%	8.0%	3.2%
	Ethnic minority group	1.4%	0.5%	9.0%	3.5%
	*P value*	*0*.*687*	*0*.*567*	*0*.*236*	*0*.*176*
*Place of residence*	Urban	0.8%	0.3%	4.3%	1.3%
	Rural	1.7%	0.7%	11.4%	4.9%
	*P value*	*0*.*001*	*<0*.*001*	*<0*.*001*	*<0*.*001*
*Education*	Up to primary school	2.0%	0.8%	12.6%	5.1%
	Junior middle school	1.0%	0.4%	6.4%	2.6%
	Senior middle school	0.6%	0.3%	3.5%	1.5%
	Higher education	0.9%	0.3%	4.0%	1.2%
	*P value*	*<0*.*001*	*0*.*004*	*<0*.*001*	*<0*.*001*
*Income*	1st tertile (Low)	2.9%	1.2%	20.0%	8.2%
	2nd tertile (Medium)	0.7%	0.2%	4.1%	1.3%
	3rd tertile (High)	0.2%	0.1%	1.0%	0.4%
	*P value*	*<0*.*001*	*<0*.*001*	*<0*.*001*	*<0*.*001*
*Household size*	1~2 members	2.9%	1.3%	15.9%	6.9%
	3~4 members	0.9%	0.4%	5.3%	2.3%
	5+ members	1.1%	0.3%	6.8%	1.6%
	*P value*	*<0*.*001*	*<0*.*001*	*<0*.*001*	*<0*.*001*
*Dental*	Not insured	1.5%	0.6%	9.3%	3.9%
*Insurance*	Insured	0.7%	0.1%	3.2%	0.5%
	*P value*	*0*.*003*	*<0*.*001*	*<0*.*001*	*<0*.*001*
*Pain in teeth or*	Never	0.5%	0.2%	8.1%	4.2%
*Mouth*	Rarely	1.3%	0.5%	5.7%	2.0%
	Sometimes	1.9%	0.7%	6.6%	2.5%
	Often	5.0%	1.9%	14.5%	5.6%
	*P value*	*<0*.*001*	*<0*.*001*	*<0*.*001*	*<0*.*001*
*Number of teeth*	No teeth	3.0%	1.6%	25.4%	13.2%
	1–19 teeth	3.2%	1.2%	14.6%	5.4%
	20+ teeth	1.1%	0.4%	6.7%	2.7%
* *	*P value*	*0*.*586*	*0*.*492*	*<0*.*001*	*<0*.*001*

^a^ All p values were obtained from simple binary logistic regression models.

Tables 3 and 4 show the individual- and province-level factors associated with CDHE among all participants and service users. In the full sample, every individual-level factor, except ethnicity, was associated with CDHE in unadjusted models; and, regardless of the income threshold, used to define CDHE. However, only education, income, household size, pain in teeth or mouth and number of teeth were associated with CDHE in the adjusted model. CDHE was more likely to occur in poorer and smaller households, adults who experienced pain in their teeth or mouth, and those with fewer teeth. The association between education and CDHE changed from negative to positive after adjustments (especially for income), indicating that more educated participants were more likely to incur in CDHE. GDP per capita was the only province-level factor associated with CDHE and only at the 10% threshold. A similar pattern of results was found amongst service users. Although all individual-level factors except sex and ethnicity were associated with CDHE in the unadjusted models, only income, household size, pain in teeth or mouth and number of teeth were associated with CDHE at both thresholds. Education and GDP per capita were both positively associated with CDHE but only at the lower threshold.

**Table 3:** Individual- and province-level factors associated with catastrophic dental health expenditure at 10%- and 20%-income thresholds among Chinese adults (n = 31,566)

Factors		At 10%		At 20%	
		Unadjusted OR^a^ [95% CI]	Adjusted OR^a^ [95% CI]	Unadjusted OR^a^ [95% CI]	Adjusted OR^a^ [95% CI]
**Level 1: 31566 adults**					
*Sex*	Men	1.00 [Reference]	1.00 [Reference]	1.00 [Reference]	1.00 [Reference]
	Women	1.35 [1.10–1.66]**	1.12 [0.90–1.38]	1.45 [1.05–2.01]*	1.19 [0.85–1.68]
*Age*	35~44 years	1.00 [Reference]	1.00 [Reference]	1.00 [Reference]	1.00 [Reference]
	65~74 years	2.32 [1.90–2.84]***	1.03 [0.76–1.39]	2.50 [1.81–3.45]***	1.04 [0.63–1.70]
*Ethnicity*	Han	1.00 [Reference]	1.00 [Reference]	1.00 [Reference]	1.00 [Reference]
	Ethnic minority group	1.08 [0.76–1.52]	0.93 [0.65–1.39]	1.51 [0.93–2.46]	1.34 [0.81–2.22]
*Place of*	Urban	1.00 [Reference]	1.00 [Reference]	1.00 [Reference]	1.00 [Reference]
*residence*	Rural	2.21 [1.78–2.75]***	1.03 [0.79–1.32]	2.54 [1.78–3.62]***	1.22 [0.81–1.84]
*Education*	Up to primary school	1.00 [Reference]	1.00 [Reference]	1.00 [Reference]	1.00 [Reference]
	Junior middle school	0.49 [0.38–0.62]***	1.10 [0.83–1.47]	0.39 [0.26–0.60]***	0.94 [0.58–1.53]
	Senior middle school	0.38 [0.27–0.53]***	1.54 [1.04–2.27]*	0.42 [0.26–0.69]***	1.91 [1.05–3.46]*
	Higher education	0.32 [0.20–0.50]***	2.39 [1.39–4.10]**	0.29 [0.13–0.61]**	2.55 [1.06–6.14]*
*Income*	1st tertile (Low)	1.00 [Reference]	1.00 [Reference]	1.00 [Reference]	1.00 [Reference]
	2nd tertile (Medium)	0.22 [0.16–0.28]***	0.24 [0.18–0.31]***	0.20 [0.13–0.31]***	0.23 [0.14–0.37]***
	3rd tertile (High)	0.07 [0.05–0.11]***	0.07 [0.04–0.12]***	0.07 [0.03–0.14]***	0.08 [0.03–0.18]***
*Household*	1~2 members	1.00 [Reference]	1.00 [Reference]	1.00 [Reference]	1.00 [Reference]
*size*	3~4 members	0.31 [0.25–0.39]***	0.47 [0.35–0.63]***	0.29 [0.21–0.42]***	0.47 [0.29–0.75]**
	5+ members	0.48 [0.37–0.63]***	0.52 [0.38–0.71]***	0.42 [0.27–0.65]***	0.46 [0.28–0.74]**
*Dental*	Not insured	1.00 [Reference]	1.00 [Reference]	1.00 [Reference]	1.00 [Reference]
*insurance*	Insured	0.42 [0.30–0.60]***	0.91 [0.62–1.34]	0.35 [0.19–0.63]***	0.77 [0.40–1.49]
*Pain in*	Never	1.00 [Reference]	1.00 [Reference]	1.00 [Reference]	1.00 [Reference]
*teeth or*	Rarely	3.08 [2.16–4.39]***	3.68 [2.55–5.30]***	2.06 [1.20–3.54]**	2.43 [1.38–4.26]**
*mouth*	Sometimes	4.95 [3.63–6.76]***	5.19 [3.76–7.16]***	3.24 [2.03–5.17]***	3.29 [2.03–5.33]***
	Often	12.11 [8.83–16.60]***	10.62 [7.63–14.77]***	9.07 [5.74–14.32]***	7.52 [4.65–12.13]***
*Number of*	No teeth	1.00 [Reference]	1.00 [Reference]	1.00 [Reference]	1.00 [Reference]
*teeth*	1–19 teeth	0.82 [0.51–1.33]	0.48 [0.29–0.81]**	0.89 [0.43–1.83]	0.60 [0.27–1.32]
	20+ teeth	0.30 [0.19–0.46]***	0.28 [0.16–0.46]***	0.27 [0.13–0.53]***	0.30 [0.14–0.65]**
**Level 2: 30 Provinces**					
*GDP per capita*, *thousand yuan*		1.00 [0.98–1.01]	1.02 [1.00–1.04]*	0.99 [0.96–1.02]	1.02 [0.99–1.05]
*Gini co-efficient*, *%*		1.07 [0.98–1.16]	1.07 [0.99–1.16]	1.10 [0.97–1.24]	1.10 [0.97–1.24]
*Public health expenditure*, *%*		0.96 [0.77–1.19]	0.98 [0.80–1.20]	0.95 [0.67–1.34]	0.95 [0.69–1.31]

* p<0.05

** p<0.01

*** p<0.001

^a^ Multilevel binary logistic regression was fitted and odds ratios (OR) reported. Regression models included all factors presented in the table as explanatory variables.

**Table 4:** Factors associated with catastrophic dental health expenditure at 10%- and 20%-income thresholds among Chinese adults who visited the dentist in the last year (n = 5,511)

Factors		At 10%		At 20%	
		Unadjusted OR^a^ [95% CI]	Adjusted OR^a^ [95% CI]	Unadjusted OR^a^[95% CI]	Adjusted OR^a^ [95% CI]
**Level 1: 5511 adults**					
*Sex*	Men	1.00 [Reference]	1.00 [Reference]	1.00 [Reference]	1.00 [Reference]
	Women	1.20 [0.98–1.48]	1.19 [0.94–1.51]	1.30 [0.95–1.80]	1.24 [0.87–1.76]
*Age*	35~44 years	1.00 [Reference]	1.00 [Reference]	1.00 [Reference]	1.00 [Reference]
	65~74 years	1.97 [1.60–2.42]***	0.95 [0.69–1.31]	2.03 [1.47–2.79]	0.99 [0.61–1.63]
*Ethnicity*	Han	1.00 [Reference]	1.00 [Reference]	1.00 [Reference]	1.00 [Reference]
	Ethnic minority group	1.29 [0.91–1.81]	0.98 [0.66–1.46]	1.85 [1.15–2.97]*	1.48 [0.86–2.53]
*Place of*	Urban	1.00 [Reference]	1.00 [Reference]	1.00 [Reference]	1.00 [Reference]
*residence*	Rural	3.30 [2.65–4.12]***	1.19 [0.90–1.57]	3.63 [2.56–5.15]***	1.47 [0.94–2.27]
*Education*	Up to primary school	1.00 [Reference]	1.00 [Reference]	1.00 [Reference]	1.00 [Reference]
	Junior middle school	0.44 [0.34–0.57]***	0.86 [0.63–1.17]	0.37 [0.24–0.57]***	0.77 [0.47–1.25]
	Senior middle school	0.29 [0.21–0.40]***	1.21 [0.80–1.81]	0.34 [0.21–0.55]***	1.47 [0.79–2.71]
	Higher education	0.19 [0.12–0.30]***	1.80 [1.01–3.18]*	0.19 [0.09–0.39]***	1.86 [0.76–4.50]
*Income*	1st tertile (Low)	1.00 [Reference]	1.00 [Reference]	1.00 [Reference]	1.00 [Reference]
	2nd tertile (Medium)	0.16 [0.12–0.21]***	0.15 [0.11–0.21]***	0.16 [0.10–0.25]***	0.17 [0.11–0.28]***
	3rd tertile (High)	0.04 [0.03–0.06]***	0.04 [0.02–0.06]***	0.04 [0.02–0.09]***	0.05 [0.02–0.11]***
*Household*	1~2 members	1.00 [Reference]	1.00 [Reference]	1.00 [Reference]	1.00 [Reference]
*size*	3~4 members	0.32 [0.25–0.41]***	0.35 [0.25–0.48]***	0.32 90.23–0.46]***	0.40 [0.25–0.64]***
	5+ members	0.54 [0.41–0.72]***	0.38 [0.27–0.53]***	0.49 [0.31–0.76]**	0.38 [0.23–0.63]***
*Dental*	Not insured	1.00 [Reference]	1.00 [Reference]	1.00 [Reference]	1.00 [Reference]
*insurance*	Insured	0.33 [0.23–0.47]***	0.97 [0.64–1.45]	0.28 [0.15–0.50]***	0.88 [0.45–1.72]
*Pain in*	Never	1.00 [Reference]	1.00 [Reference]	1.00 [Reference]	1.00 [Reference]
*teeth or*	Rarely	0.78 [0.54–1.11]	0.88 [0.59–1.32]	0.53 [0.31–0.91]*	0.58 [0.33–1.05]
*mouth*	Sometimes	0.97 [0.70–1.33]	0.96 [0.67–1.37]	0.64 [0.40–1.02]	0.59 [0.36–0.99]*
	Often	2.10 [1.51–2.91]***	1.73 [1.19–2.52]**	1.54 [0.97–2.45]	1.17 [0.70–1.96]
*Number of*	No teeth	1.00 [Reference]	1.00 [Reference]	1.00 [Reference]	1.00 [Reference]
*teeth*	1–19 teeth	0.30 [0.17–0.51]***	0.29 [0.15–0.56]***	0.33 [0.16–0.68]**	0.47 [0.20–1.07]
	20+ teeth	0.13 [0.08–0.22]***	0.21 [0.11–0.41]***	0.13 [0.07–0.26]***	0.31 [0.13–0.71]**
**Level 2: 30 Provinces**					
*GDP per capita*, *thousand yuan*		1.00 [0.98–1.02]	1.03 [1.01–1.05]**	0.99 [0.96–1.02]	1.02 [0.98–1.05]
*Gini co-efficient*, *%*		1.07 [0.97–1.17]	1.09 [0.99–1.19]	1.09 [0.95–1.26]	1.11 [0.97–1.27]
*Public health expenditure*, *%*		0.98 [0.77–1.25]	1.03 [0.82–1.29]	0.98 [0.67–1.44]	1.01 [0.71–1.43]

* p<0.05

** p<0.01

*** p<0.001

^a^ Multilevel binary logistic regression was fitted and odds ratios (OR) reported. Regression models included all factors presented in the table as explanatory variables.

## Discussion

This study shows that out-of-pocket payments for dental care put a sizeable burden on households in China. Up to 1.4% of adults and 8.1% of service users in our sample spent a large amount of their household income simply because they needed to pay for dental services or related-medication. Our findings also help characterise households more likely to face catastrophic expenditure on health if they have to pay for dental services.

Some limitations of this study need to be addressed. First, our analysis was based on cross-sectional data; and, as such, unable to test for causal relationships. Second, we used data from the 3^rd^ National Oral Health Survey, 2005, which represent the latest oral health data available and the contemporary reference in China. Furthermore, by focusing on the period where health reforms started in China, this study sets out baseline data to monitor progress in achieving financial protection. Third, our CDHE estimates were based on two questions and a 12-month recall period. There is evidence that estimates of heath spending are lower when using fewer health expenditure questions and longer recall periods [36–38]. As the survey did not include questions on total household expenditure, we used household income, as a proxy, in the denominator to estimate CDHE, which may not be responsive to the means of financing health care (savings, loans, selling assets, income transfers, etc.) [7]. Also, CHE estimates are conditional on having used health services (demand not need). Some people may forgo health care due to affordability and accessibility issues. These methodological decisions imply that our CDHE estimates may be somewhat conservative. Fourth, we only use the number of teeth as an indicator of oral health status in spite of the fact that other clinical measures (such as dental caries and periodontal disease) were also collected as part of the national survey. This was because using the latter measures would have limited our analyses to dentate people. We considered it inappropriate to exclude edentulous adults as some costly dental treatments for this group, such as dental implants and dentures, or overdentures, might lead to catastrophic spending on health.

Our findings show that certain individual and contextual factors were associated with CDHE among Chinese adults. At individual-level, poorer and smaller households were more likely to experience CDHE. Low-income families have lower capacity to pay for health care [8,9]. They would need to use a larger proportion of their available income to satisfy their demands for dental care. Wealthier adults may also buy more comprehensive health insurance, including a larger set of dental treatments, which might reduce their out-of-pocket payments for dental care. Having more people in a household could potentially prevent CDHE as there may be more earners in the family who could share the responsibility to pay for dental services. Additionally, having more family members could also provide a larger network of contacts outside the household whom they could approach in case of financial need. The association between education and CDHE reversed after adjustment for household income. This finding may reflect the fact that, given similar levels of income, people with higher levels of education are often more aware of treatment options and express their needs, including aesthetic requirements, thus often opting for more expensive dental care. Adults who experienced pain in their teeth or mouth during the last year, and those with fewer teeth, were also more likely to face CDHE. Dental pain has become an important reason for dental visits in China, with evidence that 60% of adults paid no attention to signs of dental caries if there was no pain [39]. In addition, prosthodontic services (dentures) and dental implants, the main treatment options for edentulous people, are more expensive than other types of dental treatment, which increases the odds of facing CDHE. At province-level, GDP per capita was the only contextual factor associated with CDHE and this was found at the lower income threshold only. This finding may be explained by the greater accessibility of services (mainly private clinics) and the higher costs of treatments in more economically developed areas. The floating population of workers might contribute to this issue as well, since a large number of people in China have moved to work in more developed cities, whilst their family is still living in a rural area, resulting in more temporary residents in areas with higher GDP per capita. Most of them may have lower income, and thus more at risk of experiencing CDHE if they seek dental care in the urban area where they work.

Insurance coverage was not associated with CDHE in the present study. This could be explained by two interrelated factors. The first is the low proportion of people covered by health insurance in China, around 55.9% of urban- and 21.4% of rural-residents in National Health Services Survey 2003 [40], and still less than 50% of the population overall in 2005 [19,20], suggesting that out-of-pocket payments accounted for a large proportion of health expenditure. The second is the level of co-payment required for dental treatment items among those with health insurance. Although government insurance (only for civil servants) and commercial/private insurance (only for those who can afford it) are likely to cover more items of dental treatment than other types of health insurance, they only serve a small fraction of the population. In fact, a high proportion (over 85%) of out-of-pocket payments was still required in relation to dental services, even among those with insurance [22].

The present findings have some implications for policy and further research. The demand for dental care has grown in parallel to the economic development of China in the last decade. We should seize the opportunity given by ongoing debates about universal health coverage to advocate for the inclusion of essential dental care in China [41]. The expansion of population coverage by insurance promoted by government should be matched with the addition of more insured dental care items and reduction of co-payments for dental services so as to ameliorate the impact of out-of-pocket payments and improve financial protection. An evaluation of the impact of health insurance expansion experienced in China over the last decade on CDHE is the obvious next step, once the new national survey data are released. Further studies should consider the impact of specific dental treatments, particularly those that are considered essential (disease treatment) and cosmetic.

## Conclusion

This study shows that out-of-pocket payments for dental care may put a considerable, and unnecessary, burden on household finances. Among the various individual and contextual factors evaluated in this study, household income and size as well as oral health status were associated with CDHE. Poorer and smaller households, adults with pain in their teeth or mouth and those with fewer teeth were more likely to experience CDHE.
